# In vivo evidence for the unique kinetics of evoked dopamine release in the patch and matrix compartments of the striatum

**DOI:** 10.1007/s00216-021-03300-z

**Published:** 2021-04-12

**Authors:** Andrea Jaquins-Gerstl, Kathryn M. Nesbitt, Adrian C. Michael

**Affiliations:** Department of Chemistry, Chevron Science Center, University of Pittsburgh, 219 Parkman Ave., Pittsburgh, PA 15213 USA

**Keywords:** Voltammetry, Dopamine, Patch:matrix compartments, μ-Opioid receptor

## Abstract

The neurochemical transmitter dopamine (DA) is implicated in a number of diseases states, including Parkinson’s disease, schizophrenia, and drug abuse. DA terminal fields in the dorsal striatum and core region of the nucleus accumbens in the rat brain are organized as heterogeneous domains exhibiting fast and slow kinetic of DA release. The rates of dopamine release are significantly and substantially faster in the fast domains relative to the slow domains. The striatum is composed of a mosaic of spatial compartments known as the striosomes (patches) and the matrix. Extensive literature exists on the spatial organization of the patch and matrix compartments and their functions. However, little is known about these compartments as they relate to fast and slow kinetic DA domains observed by fast scan cyclic voltammetry (FSCV). Thus, we combined high spatial resolution of FSCV with detailed immunohistochemical analysis of these architectural compartments (patch and matrix) using fluorescence microscopy. Our findings demonstrated a direct correlation between patch compartments with fast domain DA kinetics and matrix compartments to slow domain DA kinetics. We also investigated the kinetic domains in two very distinct sub-regions in the striatum, the lateral dorsal striatum (LDS) and the medial dorsal striatum (MDS). The lateral dorsal striatum as opposed to the medial dorsal striatum is mainly governed by fast kinetic DA domains. These finding are highly relevant as they may hold key promise in unraveling the fast and slow kinetic DA domains and their physiological significance.

**Graphical abstract:**

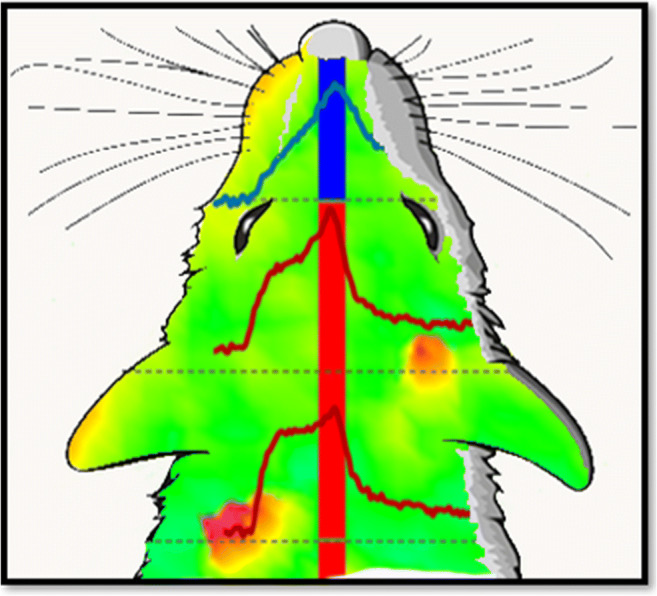

**Supplementary Information:**

The online version contains supplementary material available at 10.1007/s00216-021-03300-z.

## Introduction

Dopamine (DA) is an immensely important neurotransmitter in the central nervous system. It contributes to motor control, responses to reward, the regulation of mood and anxiety, and several other brain functions [1, 2]. Pathology of DA is clearly implicated in Parkinson’s disease, dystonia, schizophrenia, attention deficit hyperactivity disorder, and substance abuse [3–7]. Consequently, drugs that target DA have wide-ranging therapeutic applications as well as illicit uses. For these reasons, understanding brain DA activity per se and the actions of DA-targeting drugs is highly significant [8, 9]. The present study extends our previous findings, based on voltammetric recordings of electrically evoked DA release, showing that the dorsal striatum in the rat is organized as a patchwork of distinct kinetic domains [10, 11]. We have referred to these as the fast and slow domains, due to substantial differences in the patterns and initial rates of evoked DA release. The potential significance of these domains is highlighted by previous reports that the actions of several drugs, including DA antagonists and inhibitors of the DA transporter, are domain-specific [10]. We have identified autoinhibition as a contributing factor in the differential expression of kinetics in the domains of the dorsal striatum: the slow domains are tonically autoinhibited, but the fast domains are not. Our findings are consistent with abundant literature showing that presynaptic D2 autoreceptors regulate both DA release and DA uptake [12–18].

The objective of the present study was to explore the anatomical correlates of the fast and slow domains. Specifically, we wished to test the hypothesis that a correlation exists between the fast and slow kinetic domains and the well-known organization of the striatum into its patch (striosome) and matrix compartments [18–26]. At the outset of this work, we tentatively anticipated that the fast domains would correspond to patches, based simply on the observation that the fast domains, like the patch compartments, are relatively sparse in the dorsal striatum where they occupy only about 15% of the tissue [27–29]. To test this hypothesis objectively, we combined high spatial resolution voltammetric mapping of the medial and lateral portions of the dorsal striatum of the rat with detailed, post-mortem immunohistochemical analysis of the electrochemical recording sites. We based the immunohistochemical analysis on the μ-opioid receptor (MOR), a well-established marker for the patch compartment [21, 30–32].

## Materials and methods

### Carbon fiber microelectrodes

Borosilicate capillaries (0.58 mm I.D., 1.0 mm O.D., Sutter Instruments, Novato, CA) containing a carbon fiber (7-μm diameter, T650, Cytec Carbon Fibers LLC., Piedmont, SC) were pulled to a fine tip using a vertical puller (Narishige, Los Angeles, CA, USA). The tip was sealed with epoxy (Spurr Epoxy, Polysciences Inc., Warrington, PA, USA), the fiber was cut to 200 μm, and the capillary was back-filled with mercury for electrical contact to a nichrome wire (Goodfellow, Oakdale, PA) [33]. Microelectrodes were soaked for 1 h in isopropyl alcohol (Sigma Aldrich, St. Louis, MO) prior to use [34–36].

### Fast-scan cyclic voltammetry

Fast-scan cyclic voltammetry (FSCV) was performed with an EI 400 potentiostat (Ensman Instruments, Bloomington, IN) and CV Tar Heels v4.3 software. The FSCV waveform started at 0 V vs. Ag/AgCl and ramped linearly (400 V/s) to +1.0 V, −0.5 V, and 0 V [33]. The scan frequency was 10 Hz. DA was background-subtracted and quantified by integration of the oxidation current between 0.5 and 0.7 V. FSCV calibration was performed using a flow cell with N_2_-spurged artificial cerebrospinal fluid (aCSF: 142 mM NaCl, 1.2 mM CaCl_2_, 2.7 mM KCl, 1.0 mM MgCl_2_, 2.0 mM NaH_2_PO_4_, pH 7.40) containing DA (dopamine HCl, Sigma Aldrich, St. Louis, MO, USA).

### In vivo procedures

All procedures involving animals were approved by the Institutional Animal Care and Use Committee of the University of Pittsburgh. Sprague-Dawley rats (male, 250–350 g, Charles Rivers, Raleigh, NC) were anesthetized with isoflurane (induction 5%, maintenance 2.5% by volume), placed in a stereotaxic frame, and wrapped in a 37 °C heating pad (Harvard Apparatus, Holliston, MA, USA) [33]. Two microelectrodes were implanted into the striatum along with a bipolar stimulating electrode (MS303-1-untwisted, Plastics One, Roanoke, VA). One electrode was positioned over the ipsilateral medial forebrain bundle (MFB, 4.3 mm posterior to bregma, 1.2 mm lateral from bregma, and 7.2–8.5 mm below dura) and lowered to a final position that evoked DA release at both microelectrodes [33]. The electrical stimulus waveform was a biphasic, constant current, square-wave (frequency 60 Hz, pulse height 250 μA, pulse width 2 ms) supplied via an optical isolation unit (Neurolog 800, Digitimer, Herefordshire, England).

### Striatal mapping

**Fig. 1:**
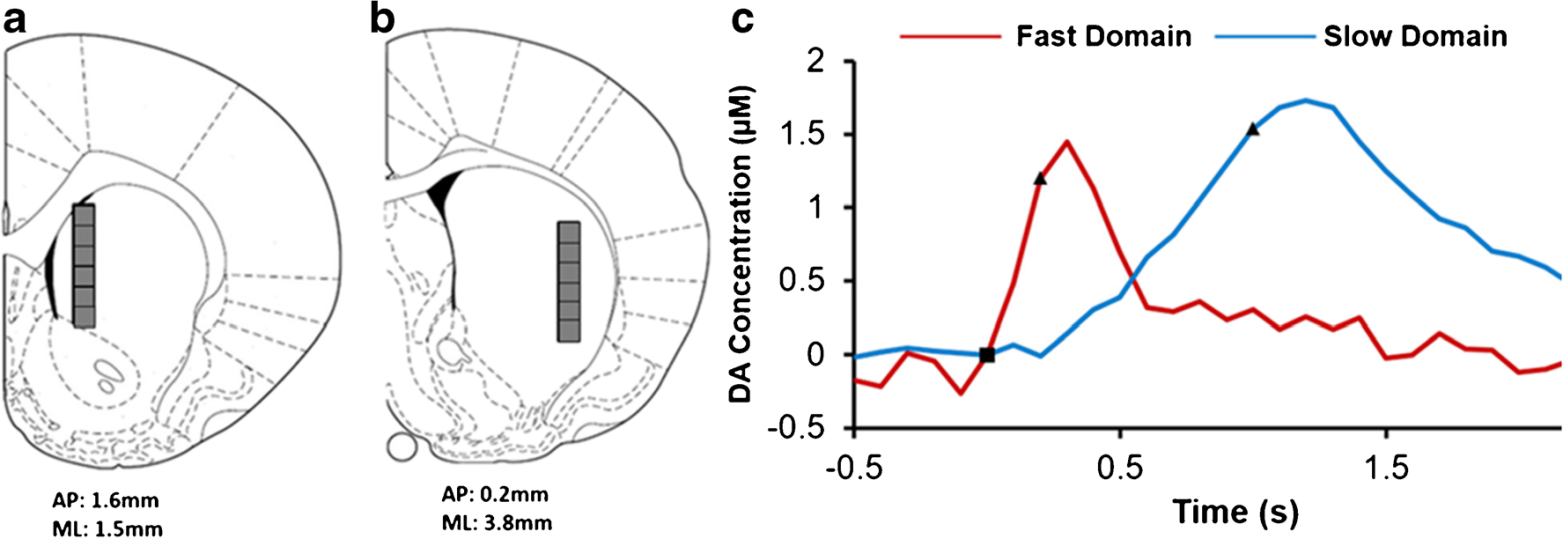
Schematic of the locations of the recording electrode tracks in the **a** MDS and **b** LDS. **c** Representative examples of fast (red) and slow (blue) evoked DA responses in the rat striatum: the symbols show when each stimulus starts (square) and stops (triangles)

We implanted two microelectrodes into the striatum of 10 individual rats (20 electrodes total). One electrode was aimed at the MDS (1.6 mm anterior to bregma, 1.5 mm lateral from bregma, and 4.5 mm below the surface) and the other at the LDS (0.2 mm anterior to bregma, 3.8 mm lateral from bregma, and 4.5 mm below the surface) for each rat [33]. Evoked DA responses were recorded along a vertical track at 6 sites of each electrode: the recording sites were 200 μm apart (total track length = 1.0 mm: total recording sites per rat = 12: total recording sites = 120). Figure 1a and b denote the location of the MDS and LDS recording tracks [33]. Each recording site was objectively classified as fast or slow by inspection of the response to a brief, 200-ms test stimulus. As we have described before [10, 11, 37–39], only fast sites respond to this brief test stimulus (Fig. 1c, red), whereas slow sites respond only to stimuli of longer duration (Fig. 1c, blue). Following the test stimulus, two further responses were recorded at each site using a stimulus duration of 1 s and 3 s [33]. After the recordings, the electrodes were lowered a further 500 μm and their final position was marked with an electrolytic lesion (35-V AC 10 s) to assist post-mortem histological localization of the recording tracks.

### Data analysis

Statistical analysis of Fig. 3 was performed by 3-way ANOVA with time (repeated measure), track, and depth as factors: time. Statistical analysis was performed with IBM SPSS software version 22.

### Tissue fixation and processing

Tissue fixation, processing, immunochemistry, and fluorescence microscopy followed our published procedures [33, 40, 41]. After the in vivo recording session, rats were perfused with PBS and paraformaldehyde. The tissue was soaked in 2% paraformaldehyde for 2 h followed by 30% sucrose overnight, frozen in liquid nitrogen-cooled 2-methylbutane, stored at −80 °C, and sliced vertically in a cryostat into 30-μm-thick sections. The sections were placed into cryoprotection solution and stored at 20 °C until immunolabeled.

### Immunofluorescence and fluorescence microscopy

Immunohistochemistry was performed using tyramide signal amplification (TSA) on free-floating sections to determine the distributional patterns of the μ-opioid receptor, MOR [33, 42–45]. Tissue sections were labeled with the primary antibody (rabbit polyclonal MOR antibody, 1:100,000; Millipore) in a 20% goat serum blocking solution at room temperature for 48 h. The secondary antibody was IgG labeled with fluorescein (1:1000; Perkin Elmer, Shelton, CT). Fluorescence microscopy (Olympus BX61, Olympus; Melville, NY) used a ×1.25 or ×10 objective and wavelength matched filter sets (Chroma Technology; Rockingham, VT). Images were analyzed and quantified with Metamorph/Fluor 7.1 (Universal Imaging Corporation; Molecular Devices), NIS Elements AR (Nikon Corporation; Tokyo, Japan), and OriginPro.

## Results

### Domain classification

**Fig. 2:**
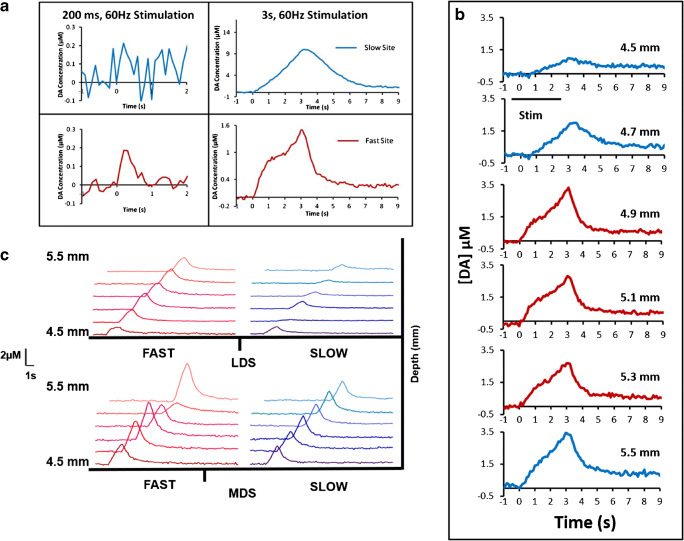
**a** Fast (red) and slow (blue) evoked DA responses recorded simultaneously with side-by-side carbon fiber microelectrodes in a single rat during the same electrical stimuli. **b** Responses from a single microelectrode track exhibiting three fast (red) and three slow (blue) sites. **c** Representative fast and slow response from all the nominal recording locations selected for this study: none of the nominal recording sites was exclusively fast or exclusively slow

Due in part to their high spatial and temporal resolution, carbon fiber microelectrodes have proven valuable in the study of central DA systems. Measurements of extracellular DA in the rat striatum during electrical stimulation of nigrostriatal DA axons in the MFB have revealed that the striatal DA terminal field forms a patchwork of distinct fast and slow kinetic domains [11, 38]. Furthermore, some DA-targeting drugs, including DA antagonists and inhibitors of the DA transporter, exhibit domain-specific actions [10, 11, 46]. In prior work [11, 38], we established an objective procedure to classify recording sites as fast or slow type based on the response to a brief test stimulus of 200 ms in duration (at 60 Hz, the test stimulus consists of 12 stimulus pulses). Only fast sites respond to this test stimulus (Fig. 2a, red). Slow sites do not respond to the test stimulus but respond robustly to longer stimuli (Fig. 2a, blue). Figure 2a reports fast and slow responses recorded simultaneously at two carbon fiber microelectrodes implanted side-by-side in a single rat during the same two stimuli. This reinforces the conclusion that the recording site determines whether the response is fast or slow type. The overall amplitude of the 3-s response from the slow site in Fig. 2a happens to be larger than that from the fast site: this emphasizes that the classification of a site as fast or slow relies on the response to the test stimulus rather than the overall response amplitude.

The microelectrodes used in this work had an active length of 200 μm (see “Materials and methods”). Therefore, each movement of the microelectrode by 200 μm along its track is presumed to establish a new recording site. Figure 2b reports a set of six responses recorded along a single microelectrode track. Based on their responses to the test stimulus, three of the responses are from fast sites (red) and three from slow sites (blue): no two responses are the same (see also Supplementary Fig. S2). Figure 2c reports representative fast and slow responses from all the nominal recording sites selected for this study (Fig. 2c combines responses from different microelectrodes and different rats). Collectively, these data confirm that each nominal recording site is unique and that none is exclusively fast or exclusively slow.

### Domains in the lateral and medial dorsal striatum

**Fig. 3:**
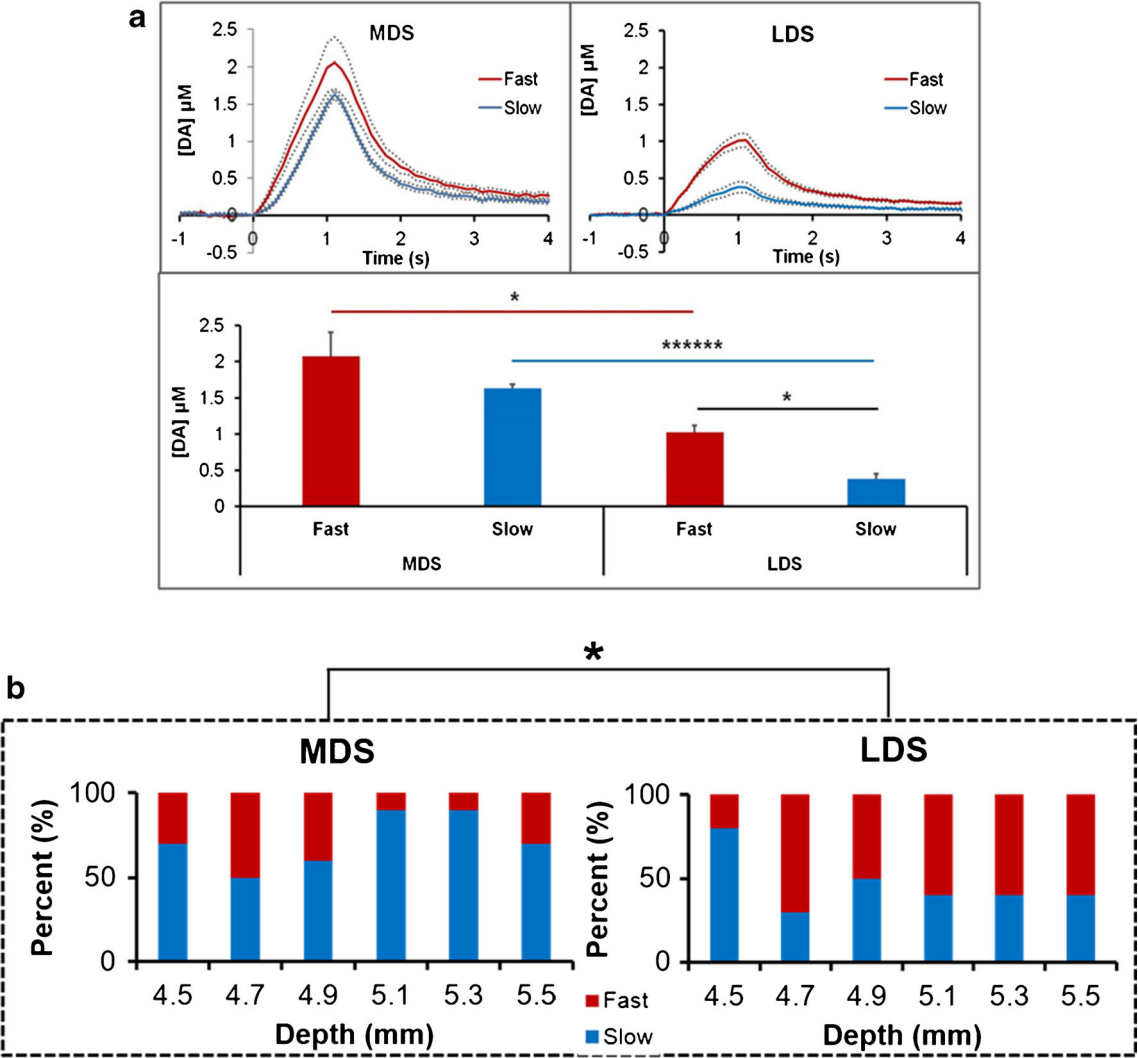
**a** Top: The overall average of fast (red) and slow (blue) evoked DA responses in the MDS and LDS (dotted lines show standard deviations). Bottom: A summary of the overall response amplitudes. Statistical analysis was by 2-way ANOVA with repeated measures with location (medial, lateral) and response type (repeated measure, fast, slow) as factors. Both location F_(1,10)_ =42.9, *p* = 0.000065, and response F_(1,10)_ = 8.65, *p* = 0.015, were significant factors. Interaction between factors was not significant. Post hoc pairwise comparisons with Bonferroni correction revealed a significant difference between fast and slow responses in the LDS only (*p < 0.05, ******p < 0.0000005). **b** The number of fast (red) and slow (blue) responses at each nominal recording site. The average number of fast responses per site in the LDS is significantly larger than that in the MDS (t-test, *p < .05)

The overall amplitude of 1-s responses from fast sites is larger than from slow sites in both the MDS and LDS (Fig. 3a). In addition, overall response amplitudes from the MDS were significantly larger than from the LDS (details of the statistical analysis are reported in the figure legend). Even though the MDS produced the larger overall response amplitudes, the MDS produced fewer fast responses than the LDS (28% and 53%, respectively: Fig. 3b). This is consistent with the point made above that the classification of fast and slow sites rests on the test stimulus rather than the overall response amplitude. Collectively, there are significant differences between the MDS and LDS in both the overall response amplitudes and the incidence of fast and slow sites.

### The correlation between domains and compartments

**Fig. 4:**
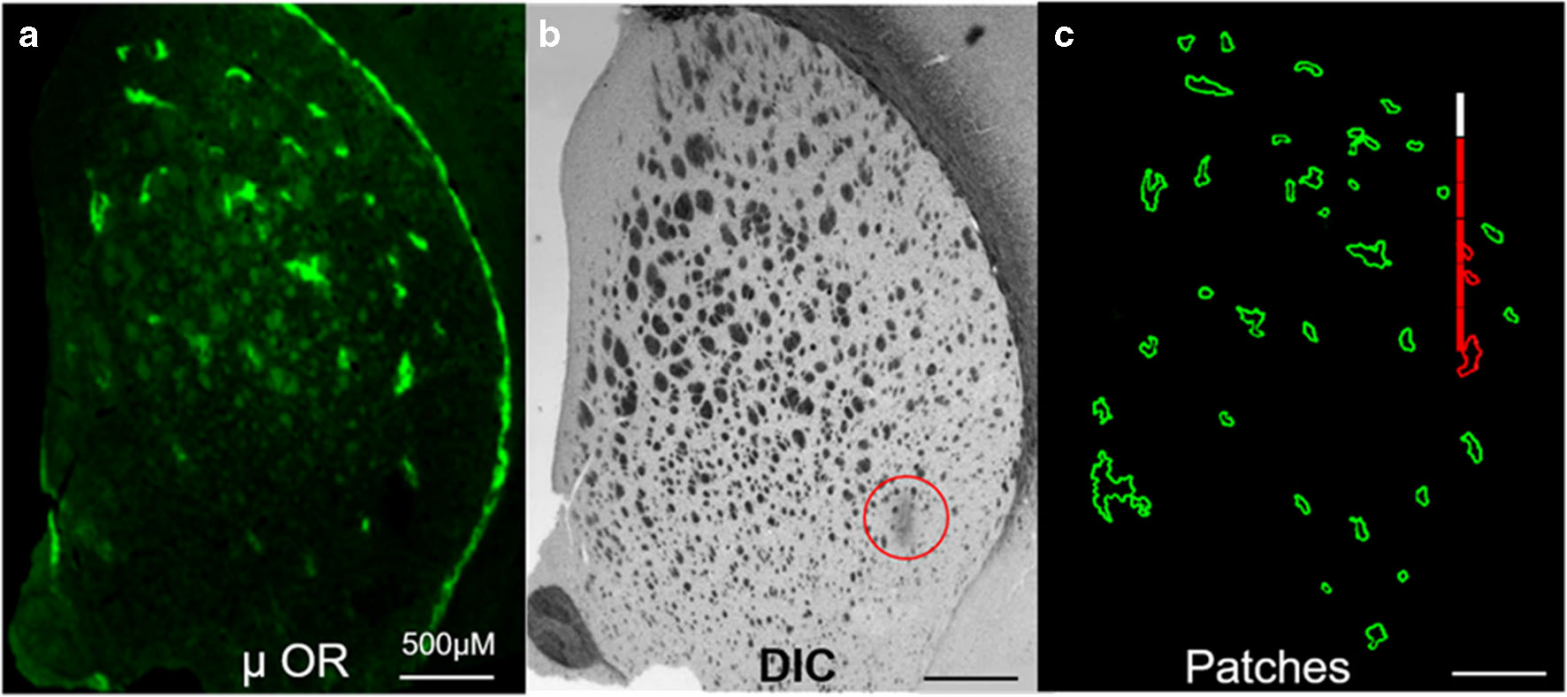
**a** Image of a striatal tissue section showing MOR immunofluorescence in green. **b** DIC image of the same section showing myelinated axon bundles and a lesion (red circle). **c** Outline drawing of the computer-identified MOR-positive patches and a scale drawing of an electrode track with fast sites in red and a slow site in white. Scale bars are 500 μm

We inspected individual recording sites by means of MOR immunofluorescence to test the hypothesis that a correlation exists between DA’s fast and slow kinetic domains and the patch and matrix compartments of the striatum. The MOR is a well-established histochemical marker for striosomes (patches). Spots of heightened MOR immunoreactivity are scattered throughout the striatal regions of all the tissue sections examined (green, Fig. 4a). A subcallosal streak of intense MOR reactivity bordering the striatum is also present, in agreement with prior studies based on [^3^H]-naloxone binding [47, 48]. MOR immunoreactivity was generally absent in other regions of the tissue, including cortical regions. Many of the less intense spots of MOR immunoreactivity are attributable to presumed non-specific labeling of myelinated axon bundles observable as dark spots in the differential interference contrast (DIC) images of the same tissue section (Fig. 4b). Therefore, the DIC and immunofluorescence images were superimposed, a fluorescence threshold intensity was set to eliminate the less intense spots of fluorescence associated with axon bundles, and spots of more intense fluorescence were outlined in the software (Fig. 4c). The computer-outlined spots are presumed to be the striatal patches (striosomes) embedded within the relatively MOR-free striatal matrix.

Based on past experience, we anticipated that the microelectrode tracks would not be visible under light microscopy [10], so we marked the bottom of each track with an electrolytic lesion. Such lesions are visible by DIC (Fig. 4b, red circle). The locations of the recording sites were estimated by measuring upwards from the lesion. Unfortunately, some of the lesions were too large to allow a precise determination of the recording site locations. Therefore, the analysis that follows is based on the subset of electrode tracks with sufficiently small lesions, which we defined as those appearing in fewer than three adjacent tissue sections (less than 90 μm). We analyzed 7 tracks in all, 2 in the LDS and 5 in MDS, with a total of 42 recording sites. The estimated locations of the recording sites along a representative track are superimposed on the outline drawing of the patches (Fig. 4c), with fast sites drawn in red and slow sites drawn in white.

**Fig. 5:**
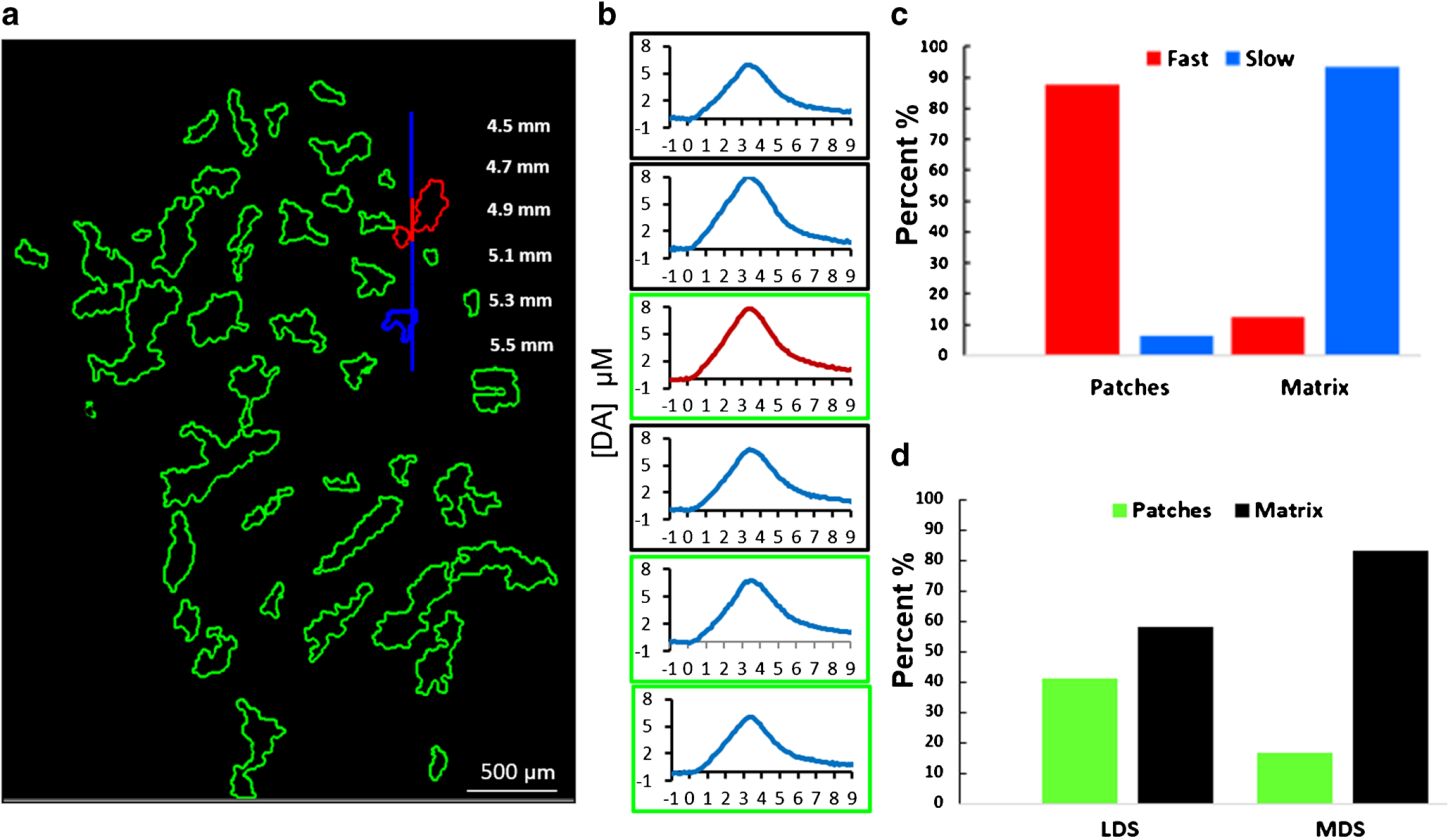
**a** Drawing of an electrode track superimposed on an outline drawing of MOR-positive patches in a striatal tissue section. The single fast site is red and the five slow sites are blue. Patches that coincide with the fast site are in red and a patch that coincides with two slows site is in blue. Scale bar is 500 μm. **b** Evoked DA responses from each recording site along the track displayed in panel a with the fast response in red and the five slow responses in blue. Responses from sites in the patch compartment are outlined in green and responses from sites in the matrix compartment are outlined in black. **c** Correlation of fast:slow sites with patch:matrix compartments, expressed as a percent. **d** Proportion of patch and matrix sites within the LDS and MDS, expressed as a percent

Figure 5 summarizes the correlation between fast and slow sites and the patch and matrix compartments. Figure 5a explains the analysis of an individual track. The fast and slow recording sites are color coded in red and blue, respectively: patches that coincide with fast sites are outlined in red (Fig. 4c also shows three red patches), while a patch that coincides with a slow site is outlined in blue. Figure 5b reports the 3-s responses from each site along the track in Fig. 5a: the response from the single fast site is coded in red and responses from the 5 slow sites are coded in blue; responses from sites that coincide with the patch compartment are outlined in green and from the matrix compartment are outlined in black. In this case, the single fast site coincides with the patch compartment, three slow sites coincide with the matrix compartment, and two slow sites coincide with the patch compartment. Figure 5c summarizes the correlation of fast and slow sites with patch and matrix compartments across all 42 sites in this analysis: fast sites predominantly (87%) coincide with the patch compartment and slow sites predominantly (93%) coincide with the matrix compartment. However, a few fast sites (13%) coincide with the matrix and a few slow sites (7%) with patches: this is discussed further in the next section.

Figure 5d reports the proportion of recording sites in the MDS and LDS identified as corresponding to the patch (green) and matrix (black) compartments. Although Fig. 5d is based on a subset of the recording sites, more recording sites in the LDS coincide with patches (40%) than in the MDS (17%). Thus, the results summarized in Figs. 3b and 5d together show that we encountered more fast sites and patches in the LDS than in the MDS.

### Proximity of fast sites to patches

**Fig. 6:**
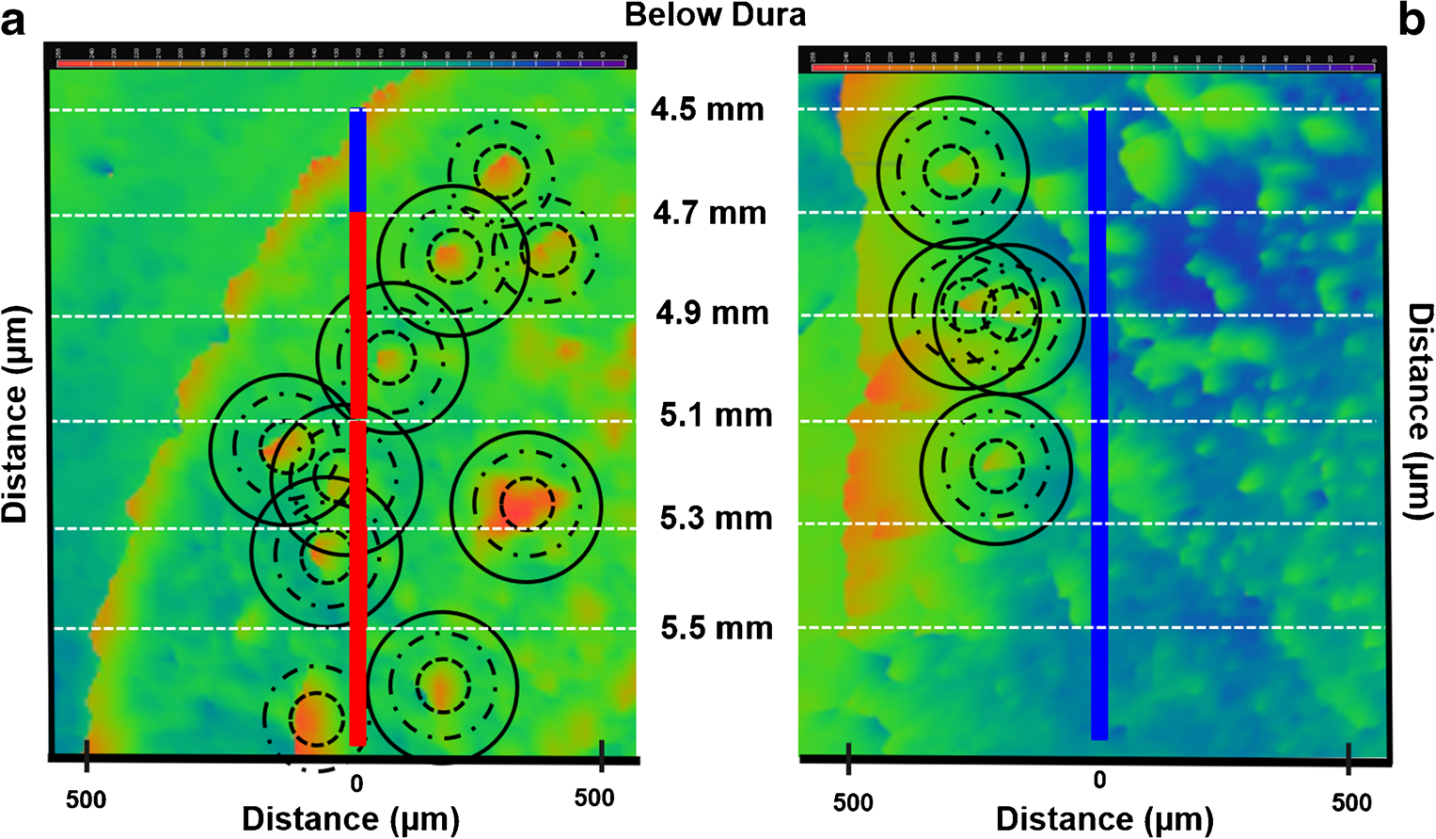
A color-coded surface intensity plot of MOR immunofluorescence in the vicinity of a microelectrode track in the **a** LDS and **b** MDS: patches and the subcallosal streak appear as spots of yellow and orange: the matrix appears mostly in green and blue (color scale is at the top of the figure). Circles with diameters of 100, 200, and 300 μm are centered on the patches. All fast sites (red) coincide with a circle. In this case, none of the slow sites coincides with any circles

The correlation between domains and compartments reported in Figs. 4 and 5 is based on a count of fast sites that coincide with a patch. However, a few fast sites (13%) did not coincide with a patch (Fig. 5b). Therefore, we wished to examine these sites in closer detail. Figure 6a is a surface intensity plot of the MOR immunofluorescence near a track in the LDS with two fast sites that did not coincide with a patch. Circles with diameters of 100, 200, and 300 μm are centered on several patches in the vicinity of the track. As in the case of this representative track, all fast sites in this study coincide with at least one of these circles. A similar analysis along a track in the MDS (Fig. 6b) that produced only slow sites shows that none of the recording sites coincides with any circles. Although this analysis does not account for the few slow sites that coincide with patches (Fig. 5c), we conclude that all the fast-recording sites in this study either coincide with or are proximal to striatal patches.

## Discussion

The findings of this study support the conclusion that the patchwork of fast and slow kinetic domains within the striatal DA terminal field correspond, respectively, to the patch and matrix striatal compartments. Across the LDS and MDS, 87% of fast sites coincided with a patch (Fig. 5) and the remaining 13% were proximal to a patch, i.e., located within 300 μm of a patch (Fig. 6). Meanwhile, 93% of slow sites coincided with the matrix and 7% coincided with a patch. Thus, fast and slow sites are predominantly associated with the patch and matrix compartments, respectively. In general terms, this conclusion supports the concept that the kinetics of DA release, diffusion, and clearance are fine-tuned to the local neurocircuitry, functional significance, and anatomical organization of the striatal brain region [49, 50].

The patch:matrix architecture of the striatum and its role in brain function and disease are well-known. For example, Graybiel and colleagues have examined how imbalances in patch and matrix properties, such as cell densities and gene expression, contribute to numerous diseases, such as Parkinson’s, Huntington’s, dystonia, and addiction [22, 51–56]. Likewise, the medial and lateral regions of the dorsal striatum, which are associated with associative and sensorimotor functions, respectively [51, 57–61], are implicated in obsessive-compulsive disorders. While the findings of the present study add to the body of information regarding the distinct properties of the patch and matrix compartments, the functional significance of the distinct DA kinetics between compartments remains to be elucidated. Further studies will be required to establish why, for example, the patches exhibit faster DA kinetics than the matrix.

It has long been conjectured that the relatively rare “hot-spots” of evoked DA release within the striatum might correspond to striatal patches, which represent the smaller and relatively sparse striatal compartment. Evidence suggests that the patch and matrix compartments receive specific projections from midbrain DA neurons [1, 62–64]. For this reason, we have wondered if the distinct fast and slow profiles are attributable to different types of midbrain DA neurons. Our studies, however, do not support this idea. Our work shows that the fast and slow profiles are readily predicted by a single mathematical model of evoked DA release kinetics, with the fast and slow responses requiring only different values of the various rate parameters [39, 65–69]. This modeling work, therefore, does not illuminate any inherent difference in the mechanisms of DA release, diffusion, and clearance that produce fast and slow responses. In light of this, we speculate that the fast and slow kinetics of evoked DA release arise from the local neurochemical environment of the DA terminals themselves. It is well-known, for example, that DA release is regulated at the pre-synaptic level by DA autoreceptors [37, 70] and heteroreceptors [1, 71–74].

We did encounter a few “mismatches” during our study: 13% of fast sites coincided with the matrix and 7% of slow sites coincided with a patch. Several factors could potentially contribute to these few mismatches. One possible factor is methodological limitations. Histological verification of carbon fiber microelectrode tracks is challenging because they are so small. During this study, we used a lesion as a histological reference mark. In several cases, however, the lesion was too large to provide a precise point of reference. Even when the lesion was sufficiently compact, accurate verification of the recording sites requires precise alignment of the tissue sections with the stereotaxic plane of the electrode tracks, which is also quite challenging. Hence, uncertainty in the location of the recording sites could have contributed to the few mismatches observed during this work.

Furthermore, there are some limitations inherent in immunohistochemical tissue analysis. First, the tissue was sectioned in the coronal plane of the electrode tracks: thus, it is possible that nearby patches in adjacent tissue sections were missed. Second, the presumed non-specific labeling of myelinated axon bundles made it necessary to threshold the images in order to identify the patches: it is possible that the thresholding procedure concealed the full extent of the patches.

We must consider the possibility that DA’s fast domains are “correlated with” but do not exactly correspond to patches, i.e., that the extent of the fast domains and the patch are not identical. This seems plausible, given that MORs are expressed by striatal neurons rather than DA terminals. Such a possibility was raised previously by Cragg [64, 68] who, by examining evoked DA release in an ex vivo striatal slice preparation, found evidence for a “peristriosomal” region, i.e., a region of unique evoked DA release in regions just surrounding striatal patches. It is possible that the fast sites we identified as “proximal” to a patch correspond to these peristriosomal regions.

As just mentioned, the conclusion that the patch and matrix striatal compartments exhibit distinct kinetics of evoked DA release is supported by prior studies in ex vivo slice preparations. A major advantage of ex vivo preparations is the greater reliability of correlating recording sites with the patch or matrix compartments. Brimblecomb and Cragg deposited a marker at the recording sites and used MOR immunofluorescence to identify patches in the same slice [64]. Salinas et al. employed a mouse strain expressing a GFP-fusion of the striosome-specific protein, Nr4a1 [69].

Nevertheless, there are interesting differences between the findings from the in vivo and ex vivo preparations. The most dramatic difference is that the ex vivo preparations do not exhibit the slow-type release dynamics observed in vivo. In the ex vivo studies, single electrical stimulus pulses evoked robust DA release at all sites, with larger responses observed in the matrix compared to the patch. This contrast is undoubtedly due to inherent differences between the in vivo and ex vivo preparations. For instance, Moquin and Michael suggested that slow-type evoked DA responses are attributable to an autoinhibitory tone of basal extracellular dopamine acting on presynaptic DA autoreceptors [11]. It appears possible that the ex vivo slice is unable to maintain this tone, perhaps due to a washout of extracellular DA, and thus does not exhibit the slow-type evoked DA profile. This is consistent with the observations of Moquin and Michael that the D2 receptor antagonist, raclopride, converts slow sites into fast sites of evoked DA release, whereas Salinas et al. report that autoinhibition does not contribute to patch:matrix differences in evoked release [69].

Another methodological difference between in vivo and ex vivo preparations might be important to consider. In our in vivo studies, the electrical stimulus was applied to ascending DA axons in the MFB, whereas the stimulus in the ex vivo preparations was applied locally and directly to the terminal field. Thus, it is likely that different neuronal circuit elements are stimulated in the two preparations. For example, Cragg and colleagues have demonstrated that cholinergic interneurons within the striatum powerfully modulate DA [71]; however, striatal interneurons would presumably not be directly activated by electrical stimulation of the midbrain. So, it might be the case the slow-type evoked DA release is specific to the axonal pathway stimulation of the in vivo preparation.

Overall, however, it is remarkable that the main conclusion, that the patch and matrix compartments exhibit distinct kinetics of evoked DA release, is mutually reinforced by studies of both the in vivo and the ex vivo preparations. This adds substantially to the robustness of the main conclusion of these collective works that the dynamics of DA transmission, and the mechanisms of actions of several DA-targeting drugs are unique to the patch and matrix compartments of the striatum.

## Supplementary information

ESM 1 (PDF 304 kb)
