# Supplementary material for: In vivo evidence for the unique kinetics of evoked dopamine release in the patch and matrix compartments of the striatum
Source: Anal Bioanal Chem. 2021 Apr 12;413(27):6703–13. doi: 10.1007/s00216-021-03300-z (PMC8551084; doi:10.1007/s00216-021-03300-z)
Supplement: Supplementary file 1 — (PDF 304 kb) [file 216_2021_3300_MOESM1_ESM.pdf]

Supplementary Information

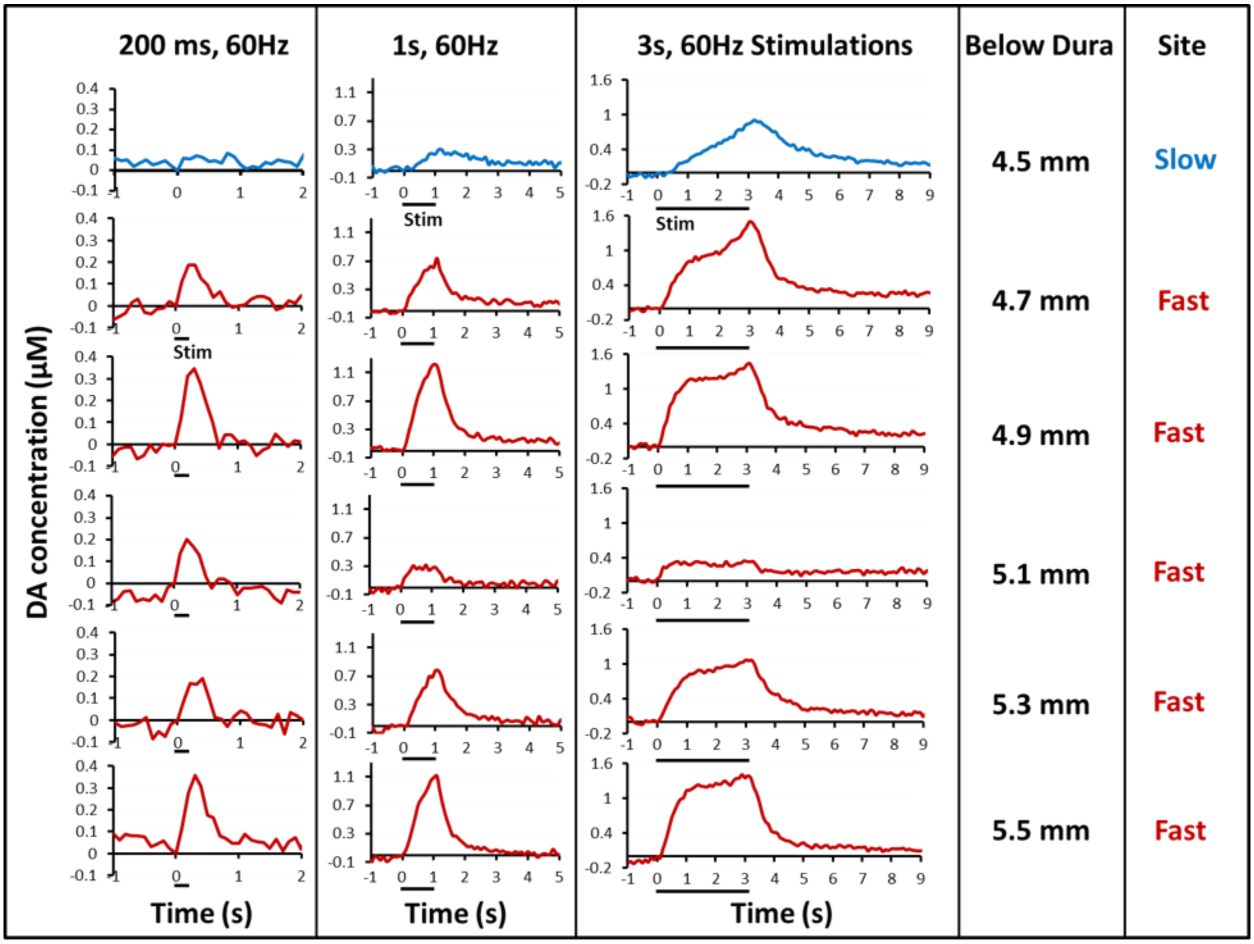

Fig. S1 A representative set of the responses recorded along a single LDS track in a single animal. At each site, three evoked responses were recorded using stimulus durations of 200 ms, 1 s, and 3 s. Each recording site was classified as either fast or slow (far right column) on the objective basis of the response to the 200 ms test stimulus: only fast sites respond to this brief test stimulus. Slow sites, however, eventually respond to the stimulus if the duration is extended beyond 200 ms. It is important to notice that the classification of fast and slow sites does not rely on the overall amplitude of the extended responses. Some fast sites, such as the one at 5.1 mm below dura in this set, give a lower overall response than some slow sites, such as the one at 4.5 mm below dura in this set

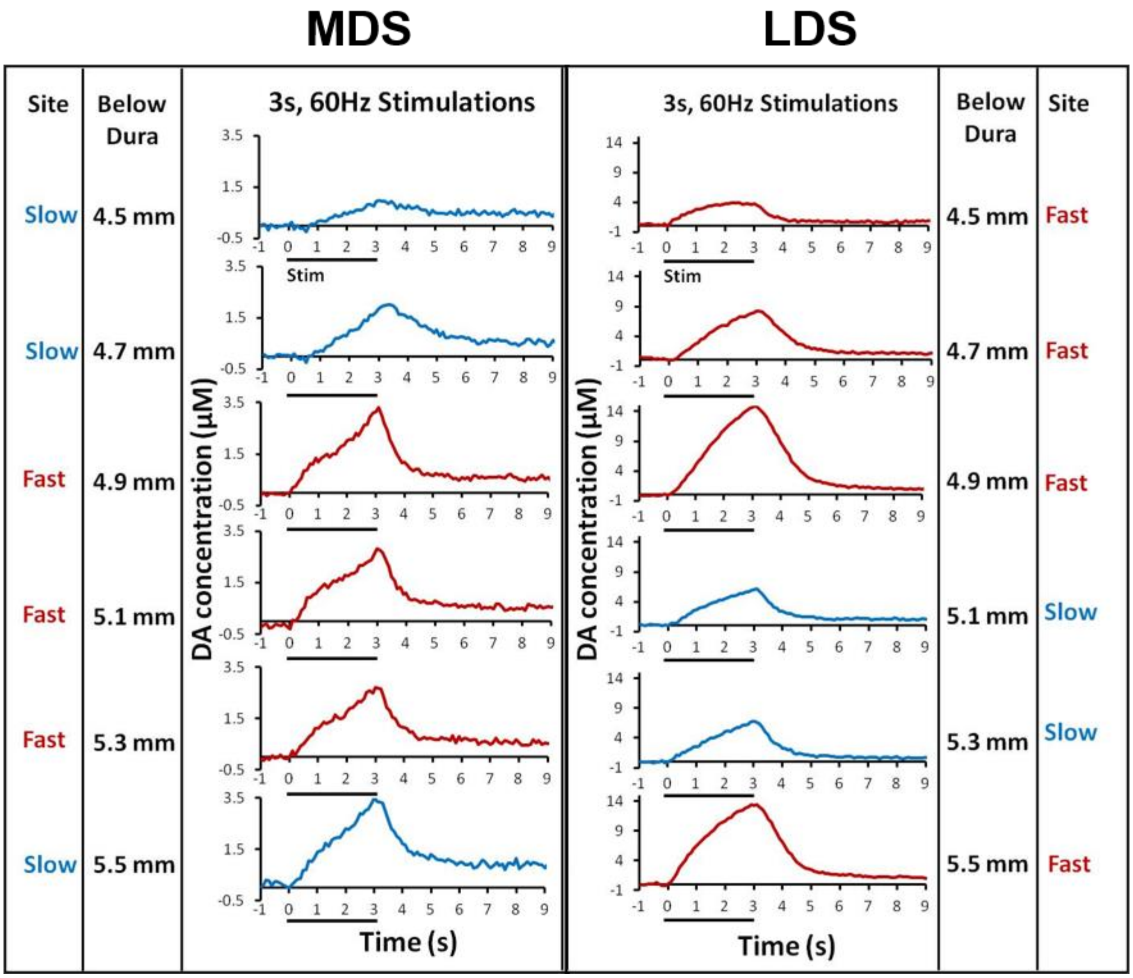

Fig. S2 A representative set of individual evoked DA responses from 12 sites along two different electrode tracks (MDS-left and LDS-right) of the same animal. Responses at each depth below dura were recorded simultaneously. Fast (red) and slow (blue) responses appeared various recording sites at both electrodes of the pair
